# Supplementary material for: Eye-tracking measures of oculomotor speed and control as markers of cognitive ability in Malawian adolescent population: Secondary analysis of a randomized controlled trial
Source: PLOS Glob Public Health. 2025 Jul 28;5(7):e0004811. doi: 10.1371/journal.pgph.0004811 (PMC12303308; doi:10.1371/journal.pgph.0004811)
Supplement: S1 Fig — After looking towards the fixation stimulus in the center of the screen, a lateral target stimulus was shown on the left or right side of the screen. The lateral target was a picture of a face in a rectangular frame. The participant was instructed to look at the lateral target as quickly as possible. Recorded xy-coordinates of gaze are shown and numbered from 1 to n samples. b) Gaze traces showing the x- and y-coordinates of gaze as a function of time for a single trial. pSRT was defined as the point at which the gaze shifted from the center of the screen to the lateral target. The x-coordinate of the borders of the central and lateral areas are shown by grey and green dashed lines. Median-filtered gaze samples are shown with dots and solid lines, raw samples as dashed lines. c) Gaze traces for all valid trials for the example observer. (DOCX) [file pgph.0004811.s001.docx]

**S1 Figure.** Prosaccade reaction time (_p_SRT) task and data. a) Illustration of a single trial in the _p_SRT task. After looking towards the fixation stimulus in the center of the screen, a lateral target stimulus was shown on the left or right side of the screen. The lateral target was a picture of a face in a rectangular frame. The participant was instructed to look at the lateral target as quickly as possible. Recorded xy-coordinates of gaze are shown and numbered from 1 to n samples. b) Gaze traces showing the x- and y-coordinates of gaze as a function of time for a single trial. _p_SRT was defined as the point at which the gaze shifted from the center of the screen to the lateral target. The x-coordinate of the borders of the central and lateral areas are shown by grey and green dashed lines. Median-filtered gaze samples are shown with dots and solid lines, raw samples as dashed lines. c) Gaze traces for all valid trials for the example observer.
