# Supplementary material for: Eye-tracking measures of oculomotor speed and control as markers of cognitive ability in Malawian adolescent population: Secondary analysis of a randomized controlled trial
Source: PLOS Glob Public Health. 2025 Jul 28;5(7):e0004811. doi: 10.1371/journal.pgph.0004811 (PMC12303308; doi:10.1371/journal.pgph.0004811)
Supplement: S2 Fig — Prosaccade error (PE) rate was estimated as a proportion of trials with a PE out of all valid trials (i.e., sum of trials with a correct aSRT, trials on which the gaze did not leave the center AOI and trials with a PE). (DOCX) [file pgph.0004811.s002.docx]

**S2 Figure.** Antisaccade reaction time (aSRT) task and data. a-b) Gaze samples on a single trial with a correct aSRT. c) Gaze samples for all valid aSRTs for one example observer, including correct aSRTs as well as trial on which there was no saccade (i.e., gaze did not leave central AOI). d-e) Gaze samples from trials with a prosaccade error. Prosaccade error (PE) rate was estimated as a proportion of trials with a PE out of all valid trials (i.e., sum of trials with a correct aSRT, trials on which the gaze did not leave the center AOI and trials with a PE).

**
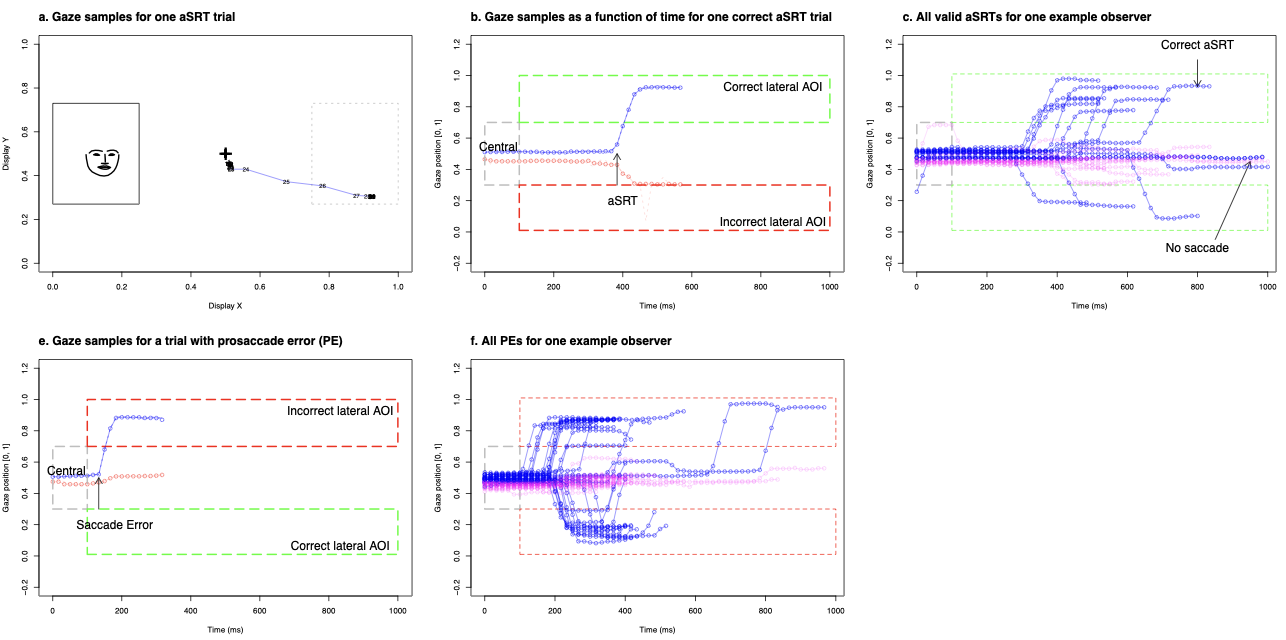
**
