# Supplementary figures and images for: Eye-tracking measures of oculomotor speed and control as markers of cognitive ability in Malawian adolescent population: Secondary analysis of a randomized controlled trial
Source: PLOS Glob Public Health. 2025 Jul 28;5(7):e0004811. doi: 10.1371/journal.pgph.0004811 (PMC12303308; doi:10.1371/journal.pgph.0004811)

**S3 Figure.** Distribution of a) mean prosaccadic rection time (pSRTm) and b) percentage of errors (PE).

**
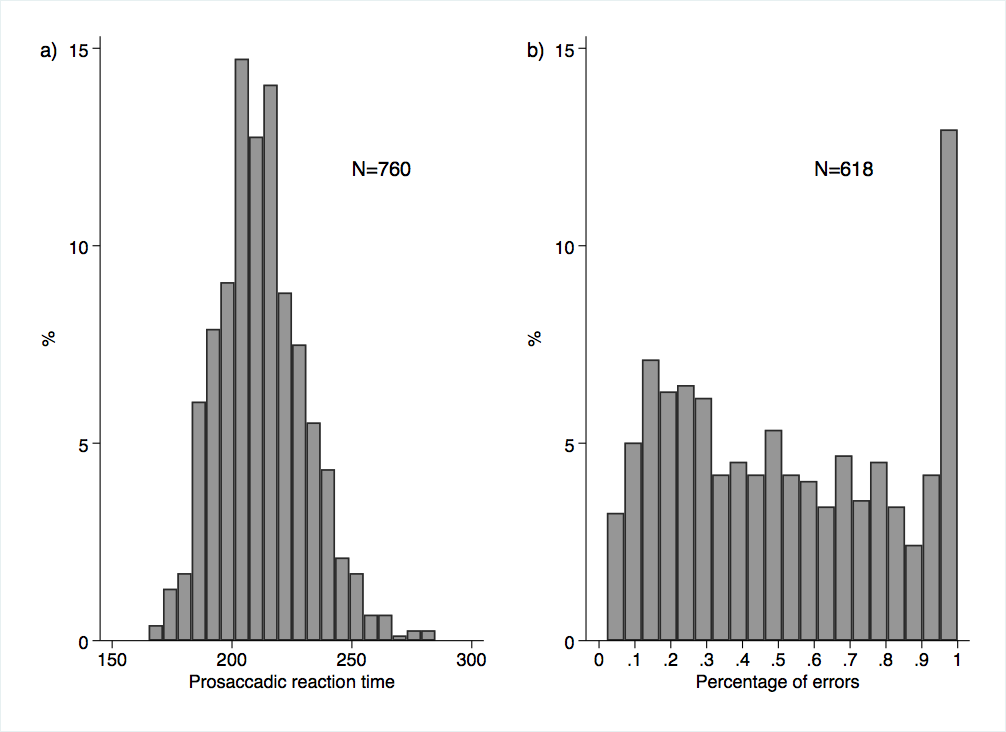
**

Supplement: S3 Fig — (DOCX) [file pgph.0004811.s003.docx]
