# Supplementary material for: Eye-tracking measures of oculomotor speed and control as markers of cognitive ability in Malawian adolescent population: Secondary analysis of a randomized controlled trial
Source: PLOS Glob Public Health. 2025 Jul 28;5(7):e0004811. doi: 10.1371/journal.pgph.0004811 (PMC12303308; doi:10.1371/journal.pgph.0004811)
Supplement: S4 Fig — Scatterplots of prosaccadic reaction time (pSRTm) and CPM score by a median split of years in school on the left. Scatterplots of PE and CPM score by median split of years in school on the right. Median split of years in school was used in the visualization, but the original continuous variable was used in the regression models. (DOCX) [file pgph.0004811.s004.docx]

**S4 Figure.** The assocation between eye tracking measures and Ravens coloured progressive matrices (CPM) score by years in school. Scatterplots of prosaccadic reaction time (_p_SRT_m_) and CPM score by a median split of years in school on the left. Scatterplots of PE and CPM score by median split of years in school on the right. Median split of years in school was used in the visualization, but the original continuous variable was used in the regression models.

**
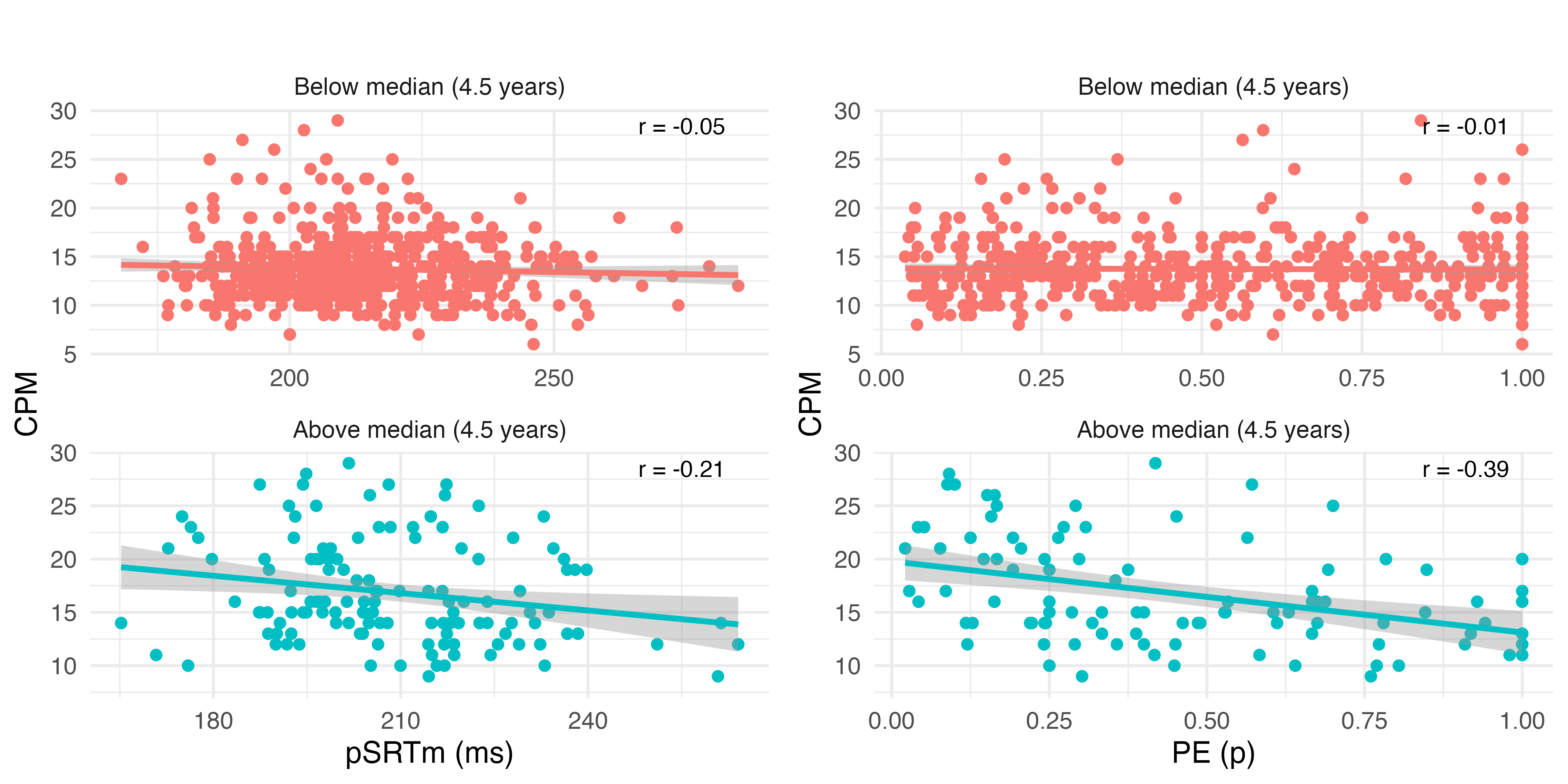
**
