# Supplementary material for: Eye-tracking measures of oculomotor speed and control as markers of cognitive ability in Malawian adolescent population: Secondary analysis of a randomized controlled trial
Source: PLOS Glob Public Health. 2025 Jul 28;5(7):e0004811. doi: 10.1371/journal.pgph.0004811 (PMC12303308; doi:10.1371/journal.pgph.0004811)
Supplement: S5 Fig — a-b) Residual plots of the unadjusted models: a) model where Raven’s coloured progressive matrices (CPM) is dependent variable and prosaccadic reaction time (SRT) independent variable, b) model where CPM is dependent variable, SRT and percentage of errors (PE) independent variables. c-d) Corresponding plots for adjusted models: c) model where CPM is dependent variable and SRT independent variable, d) model where CPM is dependent variable, SRT and PE independent variables. Models were adjusted for participant age, sex, height-for-age Z-score at 13 years, head circumference, schooling, and maternal education, the intervention during pregnancy, and socioeconomic status at 13 years. (DOCX) [file pgph.0004811.s005.docx]

**S5 Figure.** a-b) Residual plots of the unadjusted models: a) model where Raven’s coloured progressive matrices (CPM) is dependent variable and prosaccadic reaction time (SRT) independent variable, b) model where CPM is dependent variable, SRT and percentage of errors (PE) independent variables. c-d) Corresponding plots for adjusted models: c) model where CPM is dependent variable and SRT independent variable, d) model where CPM is dependent variable, SRT and PE independent variables. Models were adjusted for participant age, sex, height-for-age Z-score at 13 years, head circumference, schooling, and maternal education, the intervention during pregnancy, and socioeconomic status at 13 years.
