# Supplementary material for: Eye-tracking measures of oculomotor speed and control as markers of cognitive ability in Malawian adolescent population: Secondary analysis of a randomized controlled trial
Source: PLOS Glob Public Health. 2025 Jul 28;5(7):e0004811. doi: 10.1371/journal.pgph.0004811 (PMC12303308; doi:10.1371/journal.pgph.0004811)
Supplement: S1 Table — (DOCX) [file pgph.0004811.s007.docx]

**S1 table.** Baseline characteristics of the mothers of the included (follow-up) and excluded (lost to follow-up) participants at approximately 13 years of age

| **Characteristics** | **Follow-up (n=1003)** | **Lost to follow-up (n=324)** | **Difference (95%CI)** | **P-value** |
| --- | --- | --- | --- | --- |
| Age, years, mean (SD) | 24.9 (6.4) | 24.7 (6.4) | -0.13 (-0.94-0.68) | 0.75 |
| Gestational age at enrollment, weeks, mean (SD) | 20.1 (3.1) | 20.0 (2.9) | -0.15 (-0.54-0.23) | 0.42 |
| Proportion of primiparity | 24 % | 23 % | -0.01 (-0.06 to 0.04) | 0.712 |
| Proportion of HIV-positive participants | 11 % (N = 908) | 22 % (N =291) | 0.11 (0.06 to 0.16) | 0.000 |
| Proportion of literate participants (%) | 28.7 (0.03) | 29.3 (0.01) | -0.01 (-0.06 to 0.05) | 0.834 |
| Proportion of moderate or severe anemia, (Hb <100g/L (%)) | 26 % | 29 % | 0.03 (-0.03 to 0.08) | 0.343 |
| Years of schooling completed, mean (SD) | 2.2 (2.7) | 2.3 (2.7) | 0.02 (-0.32 to 0.36) | 0.913 |
