# Supplementary material for: Eye-tracking measures of oculomotor speed and control as markers of cognitive ability in Malawian adolescent population: Secondary analysis of a randomized controlled trial
Source: PLOS Glob Public Health. 2025 Jul 28;5(7):e0004811. doi: 10.1371/journal.pgph.0004811 (PMC12303308; doi:10.1371/journal.pgph.0004811)
Supplement: S2 Table — (DOCX) [file pgph.0004811.s008.docx]

## **S2 Table.** The association between eye-tracking results and Raven’s coloured progressive matrices score (CPM), unadjusted and adjusted models.

| Regressor | CPM | | | | | | | |
| --- | --- | --- | --- | --- | --- | --- | --- | --- |
|  | Model 1 | | Model 2 | | Model 1, adjusted | | Model 2, adjusted | |
|  | Coef. [95% CI] | P-value | Coef. [95% CI] | P-value | Coef. [95% CI] | P-value | Coef. [95% CI] | P-value |
| SRT_m_ | -0.02(-0.04- -0.005) | 0.012 | -0.02(-0.04- -0.006) | 0.008 | -0.02 (-0.03 -0.001) | 0.04 | -0.02 (-0.03 – -0.002) | 0.03 |
| PE |  |  | -1.47(-2.46 - -0.48) | 0.004 | - |  | -0.63 (-1.60 – 0.33) | 0.20 |
| Age |  |  |  |  | 0.02 (-0.004 – 0.05) | 0.09 | 0.02 (-0.007 – 0.05) | 0.14 |
| Female Sex |  |  |  |  | -0.99 (-1.63 - -0.35) | 0.003 | -0.96 (-1.60 - -0.31) | 0.004 |
| HAZ144 |  |  |  |  | -0.03 (-0.36 – 0.31) | 0.88 | -0.04 (-0.37 – 0.29) | 0.82 |
| HC144 |  |  |  |  | 0.16 (-0.06 – 0.38) | 0.14 | 0.16 (-0.06 – 0.38) | 0.15 |
| Mother’s education |  |  |  |  | 0.02 (-0.09 – 0.14) | 0.69 | 0.02 (-0.09 – 0.14) | 0.69 |
| Years of school completed |  |  |  |  | 0.56 (0.39 – 0.73) | <0.001 | 0.55 (0.37 – 0.72) | <0.001 |
| Intervention, group 2 |  |  |  |  | 0.28 (-0.41 – 0.97) | 0.38 | 0.28 (-0.40 – 1.0) | 0.42 |
| Intervention, group 3 |  |  |  |  | 0.30 (-0.41 – 1.0) | 0.42 | 0.25 (-0.46 – 0.96) | 0.48 |
| Wealth index |  |  |  |  | 0.24 (-0.06-0.56) | 0.11 | 0.27 (-0.04 – 0.57) | 0.09 |

SRT_m_=mean prosaccadic reaction time, PE= percentage of errors, HAZ144= height-for-age Z-score at 144 months, HC144= head circumference at 144 months, Intervention groups 2 and 3 compared to control group

Analysis is done including the maximal amount of the participants (with available the data required for the testing)
