# Supplementary material for: Eye-tracking measures of oculomotor speed and control as markers of cognitive ability in Malawian adolescent population: Secondary analysis of a randomized controlled trial
Source: PLOS Glob Public Health. 2025 Jul 28;5(7):e0004811. doi: 10.1371/journal.pgph.0004811 (PMC12303308; doi:10.1371/journal.pgph.0004811)
Supplement: S3 Table — (DOCX) [file pgph.0004811.s009.docx]

**S3 Table.** Summary of a regression model with prosaccadic reaction time (_p_srt_m_), years of school completed, and the interaction of _p_srt_m_ and years of school completed as predictors of Raven’s coloured progressive matrices score (CPM) score.

| Regressor | Coef. (95% CI) | P-value | Adjusted r-squared | RMSE |
| --- | --- | --- | --- | --- |
| _p_srt_m_ | 0.02 (-0.01 – 0.04) | 0.25 | 0.11 | 3.5 |
| school years | 3.0 (1.31 – 4.60) | <0.001 |  |  |
| _p_srt_m_ x school | -0.01 (-0.02 - -0.003) | 0.006 |  |  |
