# Supplementary material for: Eye-tracking measures of oculomotor speed and control as markers of cognitive ability in Malawian adolescent population: Secondary analysis of a randomized controlled trial
Source: PLOS Glob Public Health. 2025 Jul 28;5(7):e0004811. doi: 10.1371/journal.pgph.0004811 (PMC12303308; doi:10.1371/journal.pgph.0004811)
Supplement: S4 Table — (DOCX) [file pgph.0004811.s010.docx]

**Supplemental Table 4.** Summary of a regression model with percentage errors (PE), years of school completed, and the interaction of PE and years of school completed as predictors of Raven’s coloured progressive matrices score (CPM) score.

| Regressor | Coef. (95% CI) | P-value | Adjusted r-squared | RMSE |
| --- | --- | --- | --- | --- |
| PE | 2.1 (0.35 – 3.85) | 0.02 | 0.11 | 3.5 |
| school | 1.09 (0.78 – 1.39) | <0.001 |  |  |
| PE x school | -1.08 (-1.62 - -0.54) | <0.001 |  |  |
