# Supplementary material for: Eye-tracking measures of oculomotor speed and control as markers of cognitive ability in Malawian adolescent population: Secondary analysis of a randomized controlled trial
Source: PLOS Glob Public Health. 2025 Jul 28;5(7):e0004811. doi: 10.1371/journal.pgph.0004811 (PMC12303308; doi:10.1371/journal.pgph.0004811)
Supplement: S5 Table — (DOCX) [file pgph.0004811.s011.docx]

## **Supplemental Table 5.** Spearman correlation coefficients among 7 different eye-tracking tasks and Raven’s coloured progressive matrices score (CPM) at 13 years of age.

| SRT_sd_ | PE | LA_m_ | LA_sd_ | LE_m_ | LE_sd_ |  | CPM |
| --- | --- | --- | --- | --- | --- | --- | --- |
| 0.60** | -0.02 | 0.14** | 0.11* | 0.32** | 0.15** | SRT_m_ | -0.08* |
|  | -0.04 | 0.03 | 0.10* | 0.15** | 0.11* | SRT_sd_ | -0.007 |
|  |  | -0.10* | -0.10* | -0.07 | -0.07 | PE | -0.09* |
|  |  |  | 0.55** | 0.37** | 0.30** | LA_m_ | -0.02 |
|  |  |  |  | 0.21** | 0.22** | LA_sd_ | -0.002 |
|  |  |  |  |  | 0.85** | LE_m_ | -0.03 |
|  |  |  |  |  |  | LE_sd_ | -0.02 |

SRT_m_= Prosaccadic reaction time, mean, SRT_sd_= Reaction time, standard deviation, PE = percentage of errors, LA_m_=mean latency of accurate eye movement, LA_sd_=standard deviation of accurate eye movement, LE_m_=mean latency of error movement, LE_sd_=standard deviationof error eye movement, CPM=Raven’s coloured progressive matrices

N varies from 489 to 757, all the participants with data from each measurement included in the analysis.

*p<0.05

**p<0.001
