# Supplementary material for: Eye-tracking measures of oculomotor speed and control as markers of cognitive ability in Malawian adolescent population: Secondary analysis of a randomized controlled trial
Source: PLOS Glob Public Health. 2025 Jul 28;5(7):e0004811. doi: 10.1371/journal.pgph.0004811 (PMC12303308; doi:10.1371/journal.pgph.0004811)
Supplement: Attachment — Submitted filename: Response to Reviewers PGPH 2025-02-07_KV.docx [file pgph.0004811.s012.docx]

16 February 2025

Academic Editor Valentina Gallo

**PLOS Global Public Health**

**Re: PGPH-D-24-01531
Eye-tracking measures of oculomotor speed and control as markers of cognitive ability in Malawian adolescent population: Secondary analysis of a randomized controlled trial
PLOS Global Public Health**

Dear Editor Gallo,

We thank you for your response to our manuscript. Please find attached a revised version, in response to your invitation to resubmit the paper to PLOS Global Public Health. We have modified the manuscript according to the comments from the editorial office and the two reviewers. We felt that we could satisfactorily address the concerns of the reviewers.

Our response to the comments is presented using the following structure: The original editorial / referee comment is copied into this letter in italics. Our response to the comment is written below in normal text format. In case a revision to the manuscript text has been made, we give the appropriate line number(s). Changes in the text have been indicated using yellow text highlighting in the “Revised Manuscript with Track Changes”, and copied into this letter in the same format. The highlighted text file has been uploaded into your electronic system as a supplemental file.

We thank you and the external reviewers for the helpful feedback and hope that in its present form the manuscript is acceptable for publication in PLOS Global Public Health. The material has not and will not be offered elsewhere for possible publication now when it is under consideration in PLOS Global Public Health.

Best regards,

Dr. Karoliina Videman

Pediatrician, specializing child neurologist

Tampere University, Faculty of Medicine and Health Technology

E-mail: [karoliina.videman@tuni.fi](mailto:karoliina.videman@tuni.fi)

Phone: +358 503080253

**Responses to the comments made by the associate editor:**

EDITORIAL BOARD COMMENTS:

**Journal Requirements:**

1. *Please include a complete copy of PLOS’ questionnaire on inclusivity in global research in your revised manuscript. Our policy for research in this area aims to improve transparency in the reporting of research performed outside of researchers’ own country or community. The policy applies to researchers who have travelled to a different country to conduct research, research with Indigenous populations or their lands, and research on cultural artefacts. The questionnaire can also be requested at the journal’s discretion for any other submissions, even if these conditions are not met.  Please find more information on the policy and a link to download a blank copy of the questionnaire here:* [***https://journals.plos.org/plosone/s/best-practices-in-research-reporting***](https://eur04.safelinks.protection.outlook.com/?url=https%3A%2F%2Fjournals.plos.org%2Fplosone%2Fs%2Fbest-practices-in-research-reporting&data=05%7C02%7Ckaroliina.videman%40tuni.fi%7Cce3eeb069b2f42aecde708dd0b21f0a4%7Cfa6944afcc7c4cd89154c01132798910%7C0%7C0%7C638678962587608269%7CUnknown%7CTWFpbGZsb3d8eyJFbXB0eU1hcGkiOnRydWUsIlYiOiIwLjAuMDAwMCIsIlAiOiJXaW4zMiIsIkFOIjoiTWFpbCIsIldUIjoyfQ%3D%3D%7C0%7C%7C%7C&sdata=ghcPX1f5bwdU9HSPf3%2BwCK6SlBqvNTckntHRlIw%2F7AI%3D&reserved=0)***.*** *Please upload a completed version of your questionnaire as Supporting Information when you resubmit your manuscript****.***

Our response: We have included a completed PLOS’ questionnaire on Inclusivity in global research questionnaire as a supplemental file.

2*. Your current Financial Disclosure states is different from your funding information on the submission form. Please indicate by return email the full and correct funding information for your study and confirm the order in which funding contributions should appear. Please be sure to indicate whether the funders played any role in the study design, data collection and analysis, decision to publish, or preparation of the manuscript.*

Our response: Email was sent to confirm the exact funding information. Current financial disclosure is presented on the page 27 lines 557-565

This study was funded by personal grants received by KV from the Foundation for Pediatric Research in Finland; State funding for university-level health research, Tampere University Hospital, Wellbeing services county of Pirkanmaa/ Projects number 9AB082 ja 9AC097; The Finnish Medical Foundation, the Arvo and Lea Ylppö foundation and Märta Donner grant from Finnish Pediatric neurology society. The original LAIS study was supported by grants from the Academy of Finland (grants 79787 and 207010), the Foundation for Pediatric Research in Finland and the Medical Research Fund of Tampere University Hospital. The funders had no role in study design, data collection and analysis, decision to publish, or preparation of the manuscript.

3. *In the online submission form, you indicated that "Data Availability Statement: Data are available upon request due to concerns about participant confidentiality. Data requests from researchers who meet the criteria for access to confidential information can be made to Dr. Charles Mangani* ***(***[***cmangani@kuhes.ac.mw)***](mailto:cmangani@kuhes.ac.mw))*.".*

*All PLOS journals now require all data underlying the findings described in their manuscript to be freely available to other researchers, either 
1. In a public repository, 
2. Within the manuscript itself, or 
3. Uploaded as supplementary information.*

*This policy applies to all data except where public deposition would breach compliance with the protocol approved by your research ethics board. If your data cannot be made publicly available for ethical or legal reasons (e.g., public availability would compromise patient privacy), please explain your reasons by return email and your exemption request will be escalated to the editor for approval. Your exemption request will be handled independently and will not hold up the peer review process but will need to be resolved should your manuscript be accepted for publication. One of the Editorial team will then be in touch if there are any issues.*

Our response: Due to concerns about participant confidentiality the data cannot be publicly available. KV has been in contact with Peer Review Operations Specialist Carl Williams of this issue.

Data contain potentially identifying and sensitive participant information and cannot be uploaded in an open repository or shared as a supplemental file.

Data are available upon request. Data requests can be made to dean John Phuka **(edsogaph@kuhes.ac.mw)**, Kamuzu university of Health sciences. Long term data storage and availability will be assured by the study group own repository.

4. *We noticed that you used “data not shown” in the manuscript. We do not allow these references, as the PLOS data access policy requires that all data be either published with the manuscript or made available in a publicly accessible database. Please amend the supplementary material to include the referenced data or remove the references****.***

Our response: Not shown referred to one type of responses not being highlighted in Supplemental Figure 2. The responses were shown in the figure, but just not labelled. We have corrected this by adding a new figure with the missing labels. We have also removed “data not shown”.

*5. Please provide an Author Summary. This should appear in your manuscript between the Abstract (if applicable) and the Introduction, and should be 150–200 words long. The aim should be to make your findings accessible to a wide audience that includes both scientists and non-scientists. Sample summaries can be found on our website under Submission Guidelines:*

[***https://journals.plos.org/globalpublichealth/s/submission-guidelines#loc-parts-of-a-submission***](https://eur04.safelinks.protection.outlook.com/?url=https%3A%2F%2Fjournals.plos.org%2Fglobalpublichealth%2Fs%2Fsubmission-guidelines%23loc-parts-of-a-submission&data=05%7C02%7Ckaroliina.videman%40tuni.fi%7Cce3eeb069b2f42aecde708dd0b21f0a4%7Cfa6944afcc7c4cd89154c01132798910%7C0%7C0%7C638678962587621705%7CUnknown%7CTWFpbGZsb3d8eyJFbXB0eU1hcGkiOnRydWUsIlYiOiIwLjAuMDAwMCIsIlAiOiJXaW4zMiIsIkFOIjoiTWFpbCIsIldUIjoyfQ%3D%3D%7C0%7C%7C%7C&sdata=s5m47ttOCrZGb1xFaUkyiGnkbS3DdM%2FsCsi4VkPcY3o%3D&reserved=0)

Our response: Author summary including contribution has been added to page 3 lines 44-48.

**Author contributions**

Conceptualization: UA, PA, JL; Data Curation: KV; Formal Analysis: KV, LH, JL; Funding acquisition: UA, PA; Investigation: UA, CM; Methodology: UA, PA, CM, JL; Project administration: UA, CM; Supervision: UA, PA, JL; Writing – original draft: KV; Writing – review and editing: KV, UA, PA, LH, KM, CM, JL

6. *We have noticed that you have uploaded Supporting Information files, but you have not included a list of legends. Please add a full list of legends for your Supporting Information files after the references list.*

Our response: Legends have been added as requested to Supplemental Information file on page 11.

Reviewers' comments:

Reviewer's Responses to Questions

**Comments to the Author**

1. *Does this manuscript meet PLOS Global Public Health’s* [***publication criteria***](https://eur04.safelinks.protection.outlook.com/?url=https%3A%2F%2Ftrack.editorialmanager.com%2FCL0%2Fhttps%3A%252F%252Fjournals.plos.org%252Fglobalpublichealth%252Fs%252Fcriteria-for-publication%2F1%2F010f019353c18977-8cc246b7-dde0-4cd2-8f36-3f3f2d1933e4-000000%2F_rcB38YvwFSMsI6VyUgDaNabs_NoBDX6ooMGzb5jszM%3D186&data=05%7C02%7Ckaroliina.videman%40tuni.fi%7Cce3eeb069b2f42aecde708dd0b21f0a4%7Cfa6944afcc7c4cd89154c01132798910%7C0%7C0%7C638678962587637880%7CUnknown%7CTWFpbGZsb3d8eyJFbXB0eU1hcGkiOnRydWUsIlYiOiIwLjAuMDAwMCIsIlAiOiJXaW4zMiIsIkFOIjoiTWFpbCIsIldUIjoyfQ%3D%3D%7C0%7C%7C%7C&sdata=X4lYGLLdu7id%2F59ngjeUZOuiSwUVXTdlSBzlYpmmwaU%3D&reserved=0)*? Is the manuscript technically sound, and do the data support the conclusions? The manuscript must describe methodologically and ethically rigorous research with conclusions that are appropriately drawn based on the data presented.*

*Reviewer #1: Yes*

*Reviewer #2: Partly*

Our response: Thank you for your comments. We have made revisions as requested in the reviewers’ comments to make sure that the manuscript would fully meet the publication criteria.

2. *Has the statistical analysis been performed appropriately and rigorously?*

*Reviewer #1: No*

*Reviewer #2: Yes*

Our response: Please see our response to Comment 5 below. We have included additional information about our statistical analysis as requested by the reviewer.

3. *Have the authors made all data underlying the findings in their manuscript fully available (please refer to the Data Availability Statement at the start of the manuscript PDF file****)?***

*The* [***PLOS Data policy***](https://eur04.safelinks.protection.outlook.com/?url=https%3A%2F%2Ftrack.editorialmanager.com%2FCL0%2Fhttps%3A%252F%252Fjournals.plos.org%252Fglobalpublichealth%252Fs%252Fdata-availability%2F1%2F010f019353c18977-8cc246b7-dde0-4cd2-8f36-3f3f2d1933e4-000000%2FnW1DDZRkoDa2D0gLDEfGUcaONJOMTIU3xbYXnZvqgtU%3D186&data=05%7C02%7Ckaroliina.videman%40tuni.fi%7Cce3eeb069b2f42aecde708dd0b21f0a4%7Cfa6944afcc7c4cd89154c01132798910%7C0%7C0%7C638678962587654962%7CUnknown%7CTWFpbGZsb3d8eyJFbXB0eU1hcGkiOnRydWUsIlYiOiIwLjAuMDAwMCIsIlAiOiJXaW4zMiIsIkFOIjoiTWFpbCIsIldUIjoyfQ%3D%3D%7C0%7C%7C%7C&sdata=lIF5V8MPg5jNQFY%2BRovbIjWHn%2FzuZZQnqkMwdpDkLqM%3D&reserved=0) *requires authors to make all data underlying the findings described in their manuscript fully available without restriction, with rare exception. The data should be provided as part of the manuscript or its supporting information, or deposited to a public repository. For example, in addition to summary statistics, the data points behind means, medians and variance measures should be available. If there are restrictions on publicly sharing data—e.g. participant privacy or use of data from a third party—those must be specified.*

*Reviewer #1: Yes*

*Reviewer #2: Yes*

Our response: Please see our answer to the Editorial Board Comment 3 above.

4. *Is the manuscript presented in an intelligible fashion and written in standard English?

PLOS Global Public Health does not copyedit accepted manuscripts, so the language in submitted articles must be clear, correct, and unambiguous. Any typographical or grammatical errors should be corrected at revision, so please note any specific errors here.*

*Reviewer #1: Yes*

*Reviewer #2: Yes*

Our response: We thank the reviewers for the positive feedback.

5. Review Comments to the Author

*Please use the space provided to explain your answers to the questions above. You may also include additional comments for the author, including concerns about dual publication, research ethics, or publication ethics. (Please upload your review as an attachment if it exceeds 20,000 characters)*

*Reviewer #1: This is a secondary analysis of outcomes from a large Malawian adolescent study on ocular speed and cognition. The good news is that it appears to be a rich data set and power is not an issue with such large sample sizes. The authors also present reasonable specific hypotheses. Correlations and regression analyses were run. I agree with the decision to use 0.20 as a cutoff, but would have preferred Spearman to Pearson because of distributional assumptions. While the analysis seems appropriate, there are no regression diagnostics reported, such as residual plots, Q-Q plots, and other items that any introductory regression class would require of it's student projects. I don't know if distributional assumptions are appropriate or if the model accounts for heterogeneity. pal components were used to create a cognition composite, but no eigenvalue loadings are given. What is the final composite? In general, this is an interesting study with unreported information that the statistician probably has but did not report.*

*Reviewer #2: TITLE: Eye-tracking measures of oculomotor speed and control as markers of cognitive ability in Malawian adolescent population: Secondary analysis of a randomized controlled trial*

Our response: We thank the reviewer for the feedback. We now clarify our analytic approach in the Method section lines 316-319:

“The analysis plan was based on a previous study, which used least square regression models to examine the predictors of CPM in Malawian adolescents and because this approach has proven to be robust against deviations from the normality assumption with a sample size that is smaller or similar to that of the current study (39). “

We also explain on Methods section lines 319-322, that

“Residual plots and Q-Q plots for the regression models are presented in Supplemental Figure 4 and 5. Sensitivity analyses testing the associations between eye tracking and CPM score by using non-parametric Spearman correlation tests are reported in Supplemental Table 5. “

In the Results section reads as follows, we now report both Pearson and Spearman correlations for the main analyses.

For analyses examining association between eye tracking variables (lines 419-421), we report

“The results of the sensitivity testing association between eye tracking variables with Spearman correlations were similar to the Pearson correlations (Supplemental Table 5).”

Finally, we did not create cognition composite with principal component analysis. This is explained in our Methods section (236-239, 240-244, 254-255) and we have revised the description to further clarify the construction of the variables:

“The primary outcome in prosaccadic task was the mean of saccadic reaction times (_p_SRT_m_) (counted as sum of valid test prosaccadic times divided by number of valid tests), with _p_SRT_m_ defined as the time interval from the onset of the lateral target to the first entry of the gaze into the area of the lateral target (a 2.3° margin was added to the area of the target).”

“In the antisaccadic task the primary outcome was the percentage of errors (PE), defined as eye movements towards the visual target (N error/N valid trials, where N valid trials is N errors+N correct saccades + N trials without a saccade (similar to Evdokinidis et al., 2002 (36)).”

We also explain that for the CPM, “Each correct answer provides a score of 1, each incorrect or no answer is counted as 0 and the test result is the sum of the answers.”

COMMENT
*Authors should please check their study goal/aim. In line 87 – 89, “…was to further examine the informational value and utility of eye-tracking tests of processing speed and control as measures of cognitive development in low-resource settings”. Then, at the beginning of the discussion section, “…Our aim was to evaluate how eye tracking measures of speed and control are associated to CPM”*

Our response: This has been edited as suggested. See lines 102-104 in the Introduction section and line 457-458 in the Discussion section.

In the present study with 13-year-old adolescents in rural Malawian low-resource setting, our goal was to examine if the eye-tracking measures of processing speed and control are associated with non-verbal inductive reasoning, which is an established correlate of processing speed and a proxy measure for general cognitive ability (27).

Our aim was to evaluate how eye tracking measures of speed and control are associated with CPM

*Line 90 -91 “Our a priori hypotheses were that faster sensory-motor speed, as indicated by mean pro-saccadic…”. Please revise for clarity*

Our response: The revised text in the Methods section reads as follows, see lines 105-107.

More precisely, we hypothesized that faster eye-tracking measured mean pro-saccadic reaction time (_p_SRT_m_), is associated with better performance in Raven’s coloured progressive matrices test (CPM) (28,29).

*Please justify the inclusion of ‘maternal characteristics’ in Table 1, as their relevance to the current study is unclear*

Our response: We provide justification for providing this data on the Methods section, see lines 282-286.

As per our statistical analysis plan, we used some maternal information as covariates (education of mother and intervention during pregnancy). This is a longitudinal follow-up study and some of the characteristics, like hemoglobin concentration of mother and literacy, can affect cognitive development of the offspring. This earlier collected information is provided in the Table 1.

*Line 142- 143 “The study participants were approximately 13-year-old at the time of the follow-up”.
Please clarify how the participants were approximately 13 years old at the time of the follow-up, given that the original study was conducted between 2003 and 2006 among pregnant women*

Our response: We thank the reviewer for a very precise comment. The revised text in the Methods section reads as follows, see lines 142-144 and 159.

The target population comprised of pregnant women who came to the antenatal care between December 2003 and October 2006.

The current follow-up was planned as a separate wave of assessment and implemented between January 19, 2018, and March 19, 2019. The study participants were mean 12.8 years old at the time of the follow-up (age range from 10.9 years to 14.6 years).

*Regarding Discussion Section:
The discussion section requires further clarification and elaboration to strengthen its overall coherence and impact.*

Our response: We thank reviewer for the feedback. We edited the Discussion section, see lines 459-462, 468-471, 474-479, 480-483, 491-492, 496-498, 500-503, 505-506, 517-519, 528-530, 534-536

There was a weak and statistically significant correlation between eye-tracking measures of mean prosaccadic reaction time (_p_SRT_m_) and CPM in the predicted direction, although the correlation was smaller than expected not exceeding the set 0.20 cut-off.

There are limitations that might have weakened the expected associations between eye tracking measures and CPM. Measurement error may lead to a significant underestimation of the size of the true correlation between the measures (46). For this reason, it is important to consider different sources of measurement error in our main outcomes (i.e., _p_SRT_m_, PE, and CPM) and the potential contribution of this e on the examined associations. In general, measurement error may arise from variations in test conditions, measurement instruments, missing data, and measurement bias. For example, light circumstances, instrumental imprecision, experimenter-dependent causes, and in addition, test subject’s fatigue, mood, hours of sleep the previous night, etc. can cause noise to the results. Many of these factors are difficult to perfectly control among human participants and experimenters. Measurement error is typically estimated by calculating test-retest reliability estimates across repeated testings and controlled for by calculating estimates of a latent construct (47), but such data were not available in our study.

However, all children in the current study were assessed in the same room, with the same equipment, and by the same experienced assessors. With standardized operating procedures, practice trials prior to actual testing and quality forms used, we tried to assure standardized test settings and test performance for each participant.

There are variety of factors that can affect brain development and myelination among children living in low-resource settings, such as early inflammation, nutrition, nurturing care and home environment, to mention some (50)

It is, therefore, possible that variation in prosaccadic reaction time captures the effects of multiple factors in the child’s growth environment that are relevant for cognitive development. However, our results show that prosaccadic reaction time alone is not sufficiently strongly associated with cognition to have clear practical utility in low resource setting.

The performance of the antisaccadic task is regulated by many cortical and subcortical brain areas, and the ability to suppress involuntary responses is crucial for daily living (14). In addition, working memory and attention has been shown to predict antisaccadic performance (53). In earlier studies, PE has varied from 0 and 30% in adult population, and 40 to 50% in children (11,15,36). High PE is typical in the beginning of the task, and as a result of learning, there are less errors. Enduring high levels of errors have been related to ADHD (13), frontal lobe lesions (54) or mental and neurological problems (55).

Our regression models showed that after controlling for covariates, prosaccadic reaction time still weakly predicts CPM, but PE does not. This finding suggests that antisaccadic test does not contribute independent predictive value for assessing cognitive development in LMIC setting among adolescent population.

In this Malawian study, female sex was associated with poorer performance in CPM, similar to our findings (39). In a study performed in Ghana among 185 children aged 6 to 12 years, age and SES (estimated by the attendance of private or public school) were positively associated CPM (58).

However, several factors, such as fatigue (60) may contribute to CPM performance, and the use of Raven’s test as a measure of general intelligence in Sub-Saharan Africa has also been questioned (29). These factors limit the interpretation of the results.

*The positioning of ‘7 twin pregnancies’ in the figure 1 is unclear and should be revised for better clarity and visual understanding.*

Our response: Figure 1 has been edited to make it clearer, and it is uploaded as a separate file.

6. *PLOS authors have the option to publish the peer review history of their article* ***(***[***what does this mean?***](https://eur04.safelinks.protection.outlook.com/?url=https%3A%2F%2Ftrack.editorialmanager.com%2FCL0%2Fhttps%3A%252F%252Fjournals.plos.org%252Fglobalpublichealth%252Fs%252Feditorial-and-peer-review-process%2523loc-peer-review-history%2F1%2F010f019353c18977-8cc246b7-dde0-4cd2-8f36-3f3f2d1933e4-000000%2FNbeXKL2diML6CfEY8KfZsoiK9LsOPp33F6wdIyoXg14%3D186&data=05%7C02%7Ckaroliina.videman%40tuni.fi%7Cce3eeb069b2f42aecde708dd0b21f0a4%7Cfa6944afcc7c4cd89154c01132798910%7C0%7C0%7C638678962587673444%7CUnknown%7CTWFpbGZsb3d8eyJFbXB0eU1hcGkiOnRydWUsIlYiOiIwLjAuMDAwMCIsIlAiOiJXaW4zMiIsIkFOIjoiTWFpbCIsIldUIjoyfQ%3D%3D%7C0%7C%7C%7C&sdata=Duv29fQyap3BfUKU39DnMiApUNVj1tf0elMfMw0F9LU%3D&reserved=0)*). If published, this will include your full peer review and any attached files.*

 *Do you want your identity to be public for this peer review? If you choose “no”, your identity will remain anonymous but your review may still be made public.


For information about this choice, including consent withdrawal, please see our* [***Privacy Policy***](https://eur04.safelinks.protection.outlook.com/?url=https%3A%2F%2Ftrack.editorialmanager.com%2FCL0%2Fhttps%3A%252F%252Fwww.plos.org%252Fprivacy-policy%2F1%2F010f019353c18977-8cc246b7-dde0-4cd2-8f36-3f3f2d1933e4-000000%2F_XUbUosM7dgOJk6s_ZSBhxrPYWlGgnbZmQVyhAult2Q%3D186&data=05%7C02%7Ckaroliina.videman%40tuni.fi%7Cce3eeb069b2f42aecde708dd0b21f0a4%7Cfa6944afcc7c4cd89154c01132798910%7C0%7C0%7C638678962587688934%7CUnknown%7CTWFpbGZsb3d8eyJFbXB0eU1hcGkiOnRydWUsIlYiOiIwLjAuMDAwMCIsIlAiOiJXaW4zMiIsIkFOIjoiTWFpbCIsIldUIjoyfQ%3D%3D%7C0%7C%7C%7C&sdata=oPZ14zob6ptQGREhKKK8jMVjc3zJRXjlgMkSQGX5%2BGk%3D&reserved=0)***.***

*Reviewer #1: No*

*Reviewer #2: No*
